# Supplementary material for: Serum Biomarkers in Carotid Artery Disease
Source: Diagnostics (Basel). 2021 Nov 18;11(11):2143. doi: 10.3390/diagnostics11112143 (PMC8619296; doi:10.3390/diagnostics11112143)
Supplement: Supplementary file 1 [file diagnostics-11-02143-s001.zip › diagnostics-1431879 supplementary.pdf]

Supplementary

Table S1. Serum biomarkers related to clinical outputs of carotid artery disease.

Type of Biomarkers	Biomarkers	Diagnosis of Carotid Artery Disease	Plaque Vulnerability	Symptomatic Carotid Artery Disease	Future Stroke Event	Stroke Severity	Cardiovascular Mortality
**Inflammatory**	hs-CRP	[1] [2, 3]	[4] [5]		[6] [7] [8]		[9]
	PTX 3	[10] [11]	[5]				
	IL-6	[1] [12]	[4] [5]				
	TNF-α	[10]	[5] [13] [14]	[15] [16]			
**Endothelial and Cell Adhesion Biomarkers**	NGAL		[17]				
	VCAM-1		[5]				[18]
	ICAM-1	[12]					[18]
	E-selectin	[12]	[5]				
	L-selectin		[13]				
Matrix Degrading or Proteolysis	MMP-1		[14]				
	TIMP-1		[14]				
	MMP-2		[19]	[20]			
	MMP-3	[12]					
	MMP-7		[14] [19]	[15]			[15]
	MMP-9	[12]	[20] [19] [21]	[20]			[22]
	MMP-14		[19]				
Lipid Related	LDL-C	[10]			[23]		
	TC				[23]		
	triglyceride				[23]		
	ox-LDL		[24]	[21]	[25]		[26]
	HDL-C		[27] [28]			[29] [23]	
	TRL	[30]	[31] [30]				
	Lp-PLA2	[32]	[32] [8]	[33]	[34] [35]		
	apoA-I					[36] [37]	
	apoE						
	PCSK9	[38]					
**Metabolic Biomarkers**	restinin		[39]	[39]			
	adiponectin	[40]		[41] [42]			
	leptin			[42]			
	FABP4	[43]	[43]	[43]			
	homocysteine	[44] [45]			[45]		
	osteoprotegerin		[46]	[46] [47]			

Table S2. A summary of studies investigating biomarkers related to carotid artery disease diagnosis.

Study	Biomarker	Methodology	Dataset	Output
Puz et al. [2]	hs-CRP	laboratory tests, ultrasound examination, statistical analysis	65 patients with ICA stenosis > 50% (39 symptomatic) and 30 healthy	Patients with ICA stenosis had significantly higher serum concentrations and CRP values than the individuals from the control group ($p = 0.009$)
Horn et al. [3] (2009)	hs-CRP	laboratory tests, ultrasound examination, statistical analysis	subclinical and advanced (INVADE study-n = 3,092, > 55 years)	rate of both subclinical and advanced stages of atherosclerosis was higher in patients with pathological hs-CRP
Debing et al. [1] (2008)	hs-CRP, IL-6, sVCAM-1	statistical analysis, high-resolution B-mode ultrasound	180 patients with ICA stenosis, 180 age-matched and sex-matched controls.	levels of hs-CRP, sVCAM-1, and IL-6 in the CEA group were significantly higher than in the control group
Yi et al. [10] (2020)	PTX 3, TNF-a, LDL-C	computed tomography angiography (CTA), statistical analysis	206 patients with ischemic stroke	plasma levels of PTX 3, TNF-α, and low-density lipoprotein cholesterol (LDL-C) were increased significantly in the CAS group patients vs. the CAS-free
Knoflach et al. [11] (2012)	PTX 3	statistical analysis, high-resolution B-mode ultrasound	132 young men, 205 young women, 562 individuals 55 to 94 years old	PTX 3 level was independently associated with prevalent
Biscetti et al. [12] (2015)	IL-6, ICAM-1, E-selectin, MMP-3, MMP-9	genetic association study	933 individuals (344 patients with ICAS-CEA endarterectomy and 589 controls)	IL-6, ICAM-1, MMP-3, and MMP-9 gene polymorphisms were independently associated with ICAS
Kofoed et al. [30] (2002)	TRL	high-resolution B-mode ultrasound and computerized image analysis	66 controls and 323 patients with carotid artery stenosis ≥ 50%	fasting and postprandial triglyceride-rich lipoproteins are elevated in patients with carotid artery stenosis of ≥ 50%
Chan et al. [38] (2016)	PCSK-9	biochemical analysis, carotid ultrasound	95 asymptomatic subjects	serum PCSK-9 remained an independent predictor of mean carotid IMT
Saarikoski et al. [40] (2010)	adiponectin	ultrasound data on carotid IMT	2,147 young adults	low serum adiponectin concentration is independently related with increased carotid IMT early atherosclerosis

Study	Biomarker	Methodology	Dataset	Output
Holm et al. [43] (2011)	FABP4	enzyme immunoassay, statistical analyses	28 asymptomatic, 31 symptomatic, 202 patients with acute ischemic stroke	FABP4 levels were higher in patients with carotid atherosclerosis
Jia et al. [44] (2016)	homocysteine	carotid duplex ultrasound examination	5393 Chinese participants	Hcy > 19.3μmol/L was considered as an independent indicator of asymptomatic CAS
Alsulaimani et al. [45] (plaque area) (2013)	homocysteine	ultrasonographic assessment of plaque morphology measured by gray-scale median	1327 stroke-free subjects	increasing Hcy was associated with an increasing risk

Table S3. A summary of studies investigating biomarkers related to carotid atherosclerotic plaque vulnerability

Study	Biomarker	Methodology	Dataset	Output
Yamagami et al. [4] (2004).	hs-CRP, IL-6	statistical analyses	246 patients, including 80 patients with a history of stroke/TIA	IL-6 and hs-CRP were negatively correlated with carotid plaque echogenicity
Shindo et al. [5] (2014)	hs-CRP, PTX 3, IL-6, TNF-a, VCAM-1	histological analysis, statistical analysis	58 patients with carotid stenosis	vulnerable group showed upregulation of proinflammatory cytokines, endothelial activation markers and inflammation markers. and downregulation of anti-inflammatory markers
Andersson et al. [13] (2009)	TNF-a	ultrasound procedure, measurement serum markers, statistical analysis	1,016 subjects	Plaque size was also related to increased levels of TNF-alpha
Pelisek et al. [14] (2009)	TNF-a, MMP-1, TIMP-1, MMP-7	Measurement of serum markers by ELISA assays, Multiscore analysis	patients (n = 101) were classified as histologically stable (n = 37) or unstable (n = 64).	Circulating levels of MMP-1, MMP-7, TIMP-1, and TNF-α were significantly enhanced in patients with unstable plaques
Guo et al. [19] (2018)	MMP-2, MMP-7, MMP-14	carotid plaque specimens, histology and	64 patients	The mRNA levels of MMP-2, MMP-7, MMP-9 and MMP-14 were

Study	Biomarker	Methodology	Dataset	Output
		immunohistochemistry analysis		elevated in vulnerable plaques
Alvarez et al. [20] (2004)	MMP-9	histopathologic analysis, immunohistochemistry (macrophage count, T lymphocytes, activated T lymphocytes)	40 patients with carotid artery stenosis	MMP-9 was also significantly higher in the symptomatic group and in patients with unstable plaques
Eilenberg et al. [17] (2019)	NGAL, MMP-9	histological investigation, statistical analysis	83 patients with asymptomatic carotid artery stenosis	Circulating NGAL and MMP-9/NGAL are significantly increased in asymptomatic patients with vulnerable carotid atherosclerotic plaques
Sarlon-Bartoli et al. [32] (2012)	Lp-PLA2	laboratory measurements, histological assessment and immunohistochemistry of carotid plaques, statistical analyses	42 patients (neurological symptoms were present in 16, unstable plaques in 23)	Plasma Lp-PLA2 level was independently associated with unstable carotid plaques
Yang et al. [8] (2017)	Lp-PLA2	laboratory examination, carotid ultrasonography and grouping, statistics analysis	100 patients with acute anterior circulation stroke and 50 noninfarction subjects (control group)	Hs-CRP and Lp-PLA2 levels were significantly higher in vulnerable plaque group than in mixed plaque group and stable plaque group
K. Nishi et al. [24] (2002)	Ox-LDL	histopathological characteristics of plaques, immunohistochemical analysis, statistical analysis	44 patients	The ox-LDL level was significantly higher in vulnerable than stable plaques
Mathiesen et al. [28] (2001)	HDL-C	ultrasonography, statistical Analysis	216 with carotid stenosis, 223 control subjects	Low levels of HDL-C are associated with an increased risk of having echolucent, rupture-prone atherosclerotic plaques
Peters et al. [27] (2012)	HDL-C	measurement of intima-media thickness, statistical analysis	984 individuals	Low levels of HDL-C are related to echolucency of the carotid intima-media

Study	Biomarker	Methodology	Dataset	Output
Nordestgaard et al. [31] (2003)	TRL, HDL	ultrasound imaging methods, histological characterization	111 asymptomatic, 135 symptomatic patients with carotid artery stenosis, 44 ipsilateral ischaemic strokes	Vulnerable plaques are associated with elevated levels of triglyceride-rich lipoproteins and with reduced levels of HDL
Kofoed et al. [30] (2002)	TRL	high-resolution B-mode ultrasound and computerized image analysis	66 controls and 323 patients with carotid artery stenosis ≥ 50%	Fasting and postprandial triglyceride-rich lipoproteins are elevated in patients with carotid artery stenosis of ≥ 50% compared with controls
Gasbarrino et al. [39] (2016)	Restinin, Chemerin	association of circulating adipokines and carotid plaque instability	n=165 symptomatic and asymptomatic patients	Low chemerin and high resistin levels were associated with plaque instability
Holm et al. [43] (2011)	FABP4	enzyme immunoassay, statistical analyses	28 asymptomatic, 31 symptomatic, 202 acute ischemic stroke	FABP4 is linked to plaque instability in patients with carotid atherosclerosis
Davaine et al. [46] (2014)	Osteoprotegerin	OPG measurement, histological and immunological analyses, statistical analysis	73 carotid plaques (49 asymptomatic and 24 symptomatic)	Circulating OPG levels were higher in the plasma of asymptomatic patients

Table S4. A summary of studies investigating biomarkers related to symptomatic carotid artery disease.

Study	Biomarkers	Methodology	Dataset	Output
Abbas et al. [15] (2014)	MMP-7	immunohistochemistry, statistical analyses	182 consecutive patients with moderate (50–69%), 23 healthy controls	MMP-7 could contribute to plaque instability
Schneiderman et al. [16] (2012)	TNF-α	lesion analysis, statistical analysis	40 symptomatic, 38 asymptomatic patients with	TNF-α was significantly increased in

Study	Biomarkers	Methodology	Dataset	Output
			progressive stenosis	symptomatic patients
B. Alvarez et al. [20] (2004)	MMP-2, MMP-9	MMP-2 and MMP-9 measurement, statistical analysis	40 patients with carotid artery stenosis	Elevated MMP-9 concentration is associated with carotid plaque instability
Sigala et al. [21] (2010)	Ox-LDL	Immunohistochemistry, data analysis and statistics	36 patients undergoing endarterectomy, 20 controls	Ox-LDL was increased in symptomatic patients
Gasbarrino [61] (2016)	Restinin	Measurement of circulation adipokines, data analysis and statistics	165 neurologically symptomatic and asymptomatic patients	resistin levels were significantly elevated in symptomatic
A. Schiro et al. [47] (2015)	Osteopontin, Osteoprotegerin	Measurement of circulating EMPs, platelet MPs (PMPs) and inflammatory markers, statistical analysis	-	Osteopontin and osteoprotegerin were significantly elevated in the symptomatic

Table S5. A summary of studies investigating biomarkers related to future stroke event.

Study	Biomarkers	Methodology	Dataset	Output
Zhou et al. [6] (2016)	hs-CRP	Meta-analysis	2436 ischemic strokes, 655 hemorrhagic strokes from 66,560 participant	When comparing the highest with the lowest hs-CRP category, the pooled RR of ischemic strokes was 1.46
Yang et al. [8] (2017)	hs-CRP	Laboratory examination, carotid ultrasonography and grouping, statistics analysis	100 patients with acute anterior circulation stroke and 50 controls	Hs-CRP and Lp-PLA2 are among the risk factors for anterior circulation stroke

Study	Biomarkers	Methodology	Dataset	Output
Ma et al. [7] (2020)	hs-CRP, TNF-α, IL-6	Data collection, statistical analysis	288 ischemic stroke patients and 300 controls	Hs-CRP, TNF-α, and IL-6 are considered to be important markers of the body's inflammatory state in ischemic stroke
Gu et al. [23] (2019)	LDL-C, TC, triglyceride	Baseline information collection, statistical analysis	Six cohort studies in China with 267, 500 participants	TC, LDL-C, and triglyceride showed positive associations with ischemic stroke
Markstad et al. [25] (2019)	Ox-LDL	Analyses of the plaque tissue: cytokines and chemokines, measurement of sLOX-1 in blood samples, statistical analysis	202 patients undergoing carotid endarterectomy	Ox-LDL induces the release of sLOX-1 from endothelial cells and that
Oei et al. [34] (2005)	Lp-PLA2	Measurement of Lp-PLA2 activity, statistical analysis	308 coronary heart disease cases, 110 ischemic stroke cases, and a random sample of 1820 subjects	Lp-PLA2 activity is an independent predictor of ischemic stroke
Elkind et al. [35] (2009)	Lp-PLA2	Measurement of Lp-PLA2 activity, statistical analysis	467 patients	Stroke patients with Lp-PLA2 activity levels in the highest quartile had an increased risk of recurrence after first ischemic stroke
Alsulaimani et al. [45] (2013)	Homocysteine	Assessment of homocysteine, assessment of carotid	1327 stroke-free subjects	elevated homocysteine was

Study	Biomarkers	Methodology	Dataset	Output
		atherosclerosis, statistical analysis		independently associated with plaque morphology
Gorgui et al. [62] (2017)	Adiponectin	systematic review and meta-analysis	-	increased adiponectin levels were associated with an increase in risk for ischemic stroke
Gairolla et al. [63] (2017)	Adiponectin, leptin	systematic review	-	levels of adiponectin and leptin are significantly associated with ischemic stroke

Table S6. A summary of studies investigating biomarkers related to cardiovascular mortality.

Study	Biomarkers	Methodology	Dataset	Output
Mayer et al. [9] (2016)	hs-CRP	Clinical and laboratory data collection, statistical analysis	1065 patients with neurological asymptomatic carotid atherosclerosis	The risk of all-cause and cardiovascular mortality significantly increased in patients with elevated serum levels of hs-CRP
Hoke et al. [18] (2015)	VCAM-1, ICAM-1	Measurement of cellular adhesion molecules, statistical analysis	855 patients	significant association between cardiovascular mortality and ICAM-1
Abbas et al. [15] (2014)	MMP-7	Immunohistochemistry, statistical analyses	182 consecutive patients with moderate (50–69%) levels, 23 healthy controls	high plasma levels of MMP-7 were independently associated with total mortality
Zhong et al. [22] (2017)	MMP-9	Data collection, statistical analysis	3186 participants, 767 participants (24.6%) experienced major disability or died	Higher log MMP-9 was associated with death and major disability.
Wang et al. [26] (2017)	Ox-LDL	Biochemical indicators, diagnosis of stroke and	3688 patients	Patients in the highest ox-LDL

Study	Biomarkers	Methodology	Dataset	Output
		stroke subtype classification, statistical analysis		quartile had a higher risk of 1-year stroke mortality
Holm et al. [43] (2011)	FABP4	Enzyme immunoassay, statistical analyses	asymptomatic (n = 28), symptomatic (n = 31), patients with acute ischemic stroke (n = 202)	levels of FABP4 were significantly associated with total and cardiovascular mortality

Table S7: Biomarkers related with different clinical outputs of carotid artery disease in i)Symptomatic, Asymptomatic & Controls, ii) Asymptomatic, iii) Symptomatic and in iv) General population

<table>
<tr><th colspan="2">i) Symptomatic, Asymptomatic & Controls</th></tr>
<tr><td>Carotid Artery Disease Diagnosis</td><td>hs-CRP [2] [3] [1], IL-6 [1] [12], sVCAM-1 [1], ICAM-1 [12], E-selectin [12], MMP-3 [12], MMP-9 [12], TRL [30], FABP4 [43]</td></tr>
<tr><td>Carotid Atherosclerotic Plaque Vulnerability</td><td>hs-CRP [4] , IL-6 [4], TRL [31] [30], HDL [31] [28], Restinin [39], Chemerin [39], FABP4 [43], Osteoprotegerin [46], Lp-PLA2 [32] ,TNF-a [14], MMP-1 [14], TIMP-1 [14], MMP-7 [14]</td></tr>
<tr><td>Symptomatic Carotid Artery Disease</td><td>MMP-7 [15], TNF-α [16] [7], hs-CRP [7], IL-6 [7], Ox-LDL [21], Restinin [61]</td></tr>
<tr><td>Cardiovascular Mortality</td><td>MMP-7 [15], FABP4 [43]</td></tr>
<tr><th colspan="2">ii) Asymptomatic</th></tr>
<tr><td>Carotid Artery Disease Diagnosis</td><td>PCSK-9 [38], homocysteine [45]</td></tr>
<tr><td>Carotid Atherosclerotic Plaque Vulnerability</td><td>NGAL [17], MMP-9 [17]</td></tr>
<tr><td>Future Stroke Event</td><td>Homocysteine [45]</td></tr>
<tr><td>Cardiovascular mortality</td><td>hs-CRP [9]</td></tr>
<tr><th colspan="2">iii) Symptomatic patients (either with diagnosis of carotid artery disease or undergoing CEA)</th></tr>
<tr><td>Carotid Artery Disease Diagnosis</td><td>PTX 3 [10], TNF-a [10], LDL-C [10]</td></tr>
<tr><td>Carotid Atherosclerotic Plaque Vulnerability</td><td>MMP-9 [20], Lp-PLA2 [8]</td></tr>
<tr><td>Future Stroke Event.</td><td>hs-CRP [6] [8], MMP-2 [20], MMP-9 [20], Ox-LDL [25], Lp-PLA2 [34]</td></tr>
<tr><th colspan="2">iv) General population</th></tr>
<tr><td>Carotid Artery Disease Diagnosis</td><td>Adiponectin [40], homocysteine [44], PTX 3 [11]</td></tr>
<tr><td>Carotid Atherosclerotic Plaque Vulnerability</td><td>MMP-2 [19], MMP-7 [19], MMP-14 [19], Ox-LDL [24], HDL-C [27], TNF-a [13]</td></tr>
<tr><td>Future Stroke Event.</td><td>LDL-C [23], TC [23], triglycerides [23], Lp-PLA2 [35]</td></tr>
<tr><td>Cardiovascular Mortality</td><td>MMP-9 [22], Ox-LDL [26], VCAM-1 [18], ICAM-1 [18]</td></tr>
</table>

References

[1] E. Debing, E. Peeters, C. Demanet, M. De Waele, and P. Van den Brande, "Markers of inflammation in patients with symptomatic and asymptomatic carotid artery stenosis: a case-control study," *Vascular and endovascular surgery,* vol. 42, pp. 122-127, 2008.

[2] P. Puz, A. Lasek-Bal, D. Ziaja, Z. Kazibutowska, and K. Ziaja, "Inflammatory markers in patients with internal carotid artery stenosis," *Archives of medical science: AMS,* vol. 9, p. 254, 2013.

[3] C. S. Horn, R. Ilg, K. Sander, H. Bickel, C. Briesenick, B. Hemmer, *et al.*, "High-sensitivity C-reactive protein at different stages of atherosclerosis: results of the INVADE study," *Journal of neurology,* vol. 256, pp. 783-791, 2009.

[4] H. Yamagami, K. Kitagawa, Y. Nagai, H. Hougaku, M. Sakaguchi, K. Kuwabara, *et al.*, "Higher levels of interleukin-6 are associated with lower echogenicity of carotid artery plaques," *Stroke,* vol. 35, pp. 677-681, 2004.

[5] A. Shindo, H. Tanemura, K. Yata, K. Hamada, M. Shibata, Y. Umeda, *et al.*, "Inflammatory biomarkers in atherosclerosis: pentraxin 3 can become a novel marker of plaque vulnerability," *PloS one,* vol. 9, p. e100045, 2014.

[6] Y. Zhou, W. Han, D. Gong, C. Man, and Y. Fan, "Hs-CRP in stroke: a meta-analysis," *Clinica chimica acta,* vol. 453, pp. 21-27, 2016.

[7] Z. Ma, Y. Yue, Y. Luo, W. Wang, Y. Cao, and Q. Fang, "Clinical utility of the inflammatory factors combined with lipid markers in the diagnostic and prognostic assessment of ischemic stroke: Based on logistic regression models," *Journal of Stroke and Cerebrovascular Diseases,* vol. 29, p. 104653, 2020.

[8] X. Liu, G. Wu, C. Xu, Y. He, L. Shu, Y. Liu, *et al.*, "Prediction of coronary plaque progression using biomechanical factors and vascular characteristics based on computed tomography angiography," *Computer Assisted Surgery,* vol. 22, pp. 286-294, 2017.

[9] F. J. Mayer, C. J. Binder, O. F. Wagner, M. Schillinger, E. Minar, W. Mlekusch, *et al.*, "Combined effects of inflammatory status and carotid atherosclerosis: a 12-year follow-up study," *Stroke,* vol. 47, pp. 2952-2958, 2016.

[10] L. Yi, J. Tang, C. Shi, T. Zhang, J. Li, F. Guo, *et al.*, "Pentraxin 3, TNF-α, and LDL-C are associated with carotid artery stenosis in patients with ischemic stroke," *Frontiers in neurology,* vol. 10, p. 1365, 2020.

[11] M. Knoflach, S. Kiechl, A. Mantovani, I. Cuccovillo, B. Bottazzi, Q. Xu, *et al.*, "Pentraxin-3 as a marker of advanced atherosclerosis results from the Bruneck, ARMY and ARFY Studies," *PloS one,* vol. 7, p. e31474, 2012.

[12] F. Biscetti, G. Straface, G. Bertoletti, C. Vincenzoni, F. Snider, V. Arena, *et al.*, "Identification of a potential proinflammatory genetic profile influencing carotid plaque vulnerability," *Journal of Vascular Surgery,* vol. 61, pp. 374-381, 2015.

[13] J. Andersson, J. Sundström, L. Kurland, T. Gustavsson, J. Hulthe, A. Elmgren, *et al.*, "The carotid artery plaque size and echogenicity are related to different cardiovascular risk factors in the elderly: the Prospective Investigation of the Vasculature in Uppsala Seniors (PIVUS) study," *Lipids,* vol. 44, p. 397, 2009.

[14] J. Pelisek, M. Rudelius, P. Zepper, H. Poppert, C. Reeps, T. Schuster, *et al.*, "Multiple biological predictors for vulnerable carotid lesions," *Cerebrovascular Diseases,* vol. 28, pp. 601-610, 2009.

[15] A. Abbas, P. Aukrust, D. Russell, K. Krohg-Sørensen, T. Almås, D. Bundgaard, *et al.*, "Matrix metalloproteinase 7 is associated with symptomatic lesions and adverse events in patients with carotid atherosclerosis," *PloS one,* vol. 9, p. e84935, 2014.

[16] J. Schneiderman, K. Schaefer, F. D. Kolodgie, N. Savion, S. Kotev-Emeth, R. Dardik, *et al.*, "Leptin locally synthesized in carotid atherosclerotic plaques could be associated with lesion instability and cerebral emboli," *Journal of the American Heart Association,* vol. 1, p. e001727, 2012.

[17] W. Eilenberg, S. Stojkovic, A. Kaider, A. Piechota-Polanczyk, J. Nanobachvili, C. M. Domenig, *et al.*, "Neutrophil gelatinase associated lipocalin (NGAL) for identification of unstable plaques in patients with asymptomatic carotid stenosis," *European Journal of Vascular and Endovascular Surgery,* 2019.

[18] M. Hoke, M.-P. Winter, O. Wagner, M. Exner, M. Schillinger, Z. Arnold, *et al.*, "The impact of selectins on mortality in stable carotid atherosclerosis," *Thrombosis and haemostasis,* vol. 114, pp. 632-638, 2015.

[19] Z. Y. Guo, B. Zhang, Y. H. Yan, S. S. Gao, J. J. Liu, L. Xu, *et al.*, "Specific matrix metalloproteinases and calcification factors are associated with the vulnerability of human carotid plaque," *Experimental and therapeutic medicine,* vol. 16, pp. 2071-2079, 2018.

[20] B. Alvarez, C. Ruiz, P. Chacón, J. Alvarez-Sabin, and M. Matas, "Serum values of metalloproteinase-2 and metalloproteinase-9 as related to unstable plaque and inflammatory cells in patients with greater than 70% carotid artery stenosis," *Journal of vascular surgery,* vol. 40, pp. 469-475, 2004.

[21] F. Sigala, A. Kotsinas, P. Savari, K. Filis, S. Markantonis, E. K. Iliodromitis, *et al.*, "Oxidized LDL in human carotid plaques is related to symptomatic carotid disease and lesion instability," *Journal of vascular surgery,* vol. 52, pp. 704-713, 2010.

[22] C. Zhong, J. Yang, T. Xu, T. Xu, Y. Peng, A. Wang, *et al.*, "Serum matrix metalloproteinase-9 levels and prognosis of acute ischemic stroke," *Neurology,* vol. 89, pp. 805-812, 2017.

[23] X. Gu, Y. Li, S. Chen, X. Yang, F. Liu, Y. Li, *et al.*, "Association of lipids with ischemic and hemorrhagic stroke: a prospective cohort study among 267 500 Chinese," *Stroke,* vol. 50, pp. 3376-3384, 2019.

[24] K. Nishi, H. Itabe, M. Uno, K. T. Kitazato, H. Horiguchi, K. Shinno, *et al.*, "Oxidized LDL in carotid plaques and plasma associates with plaque instability," *Arteriosclerosis, thrombosis, and vascular biology,* vol. 22, pp. 1649-1654, 2002.

[25] H. Markstad, A. Edsfeldt, I. Yao Mattison, E. Bengtsson, P. Singh, M. Cavalera, *et al.*, "High levels of soluble lectinlike oxidized low-density lipoprotein receptor-1 are associated with carotid plaque inflammation and increased risk of ischemic stroke," *Journal of the American Heart Association,* vol. 8, p. e009874, 2019.

[26] A. Wang, Y. Yang, Z. Su, W. Yue, H. Hao, L. Ren, *et al.*, "Association of oxidized low-density lipoprotein with prognosis of stroke and stroke subtypes," *Stroke,* vol. 48, pp. 91-97, 2017.

[27] S. Peters, L. Lind, M. Palmer, D. Grobbee, J. Crouse III, D. O'Leary, *et al.*, "Increased age, high body mass index and low HDL-C levels are related to an echolucent carotid intima–media: the METEOR study," *Journal of internal medicine,* vol. 272, pp. 257-266, 2012.

[28] E. B. Mathiesen, K. H. Bønaa, and O. Joakimsen, "Low levels of high-density lipoprotein cholesterol are associated with echolucent carotid artery plaques: the Tromsø study," *Stroke,* vol. 32, pp. 1960-1965, 2001.

[29] P.-S. Yeh, C.-M. Yang, S.-H. Lin, W.-M. Wang, P.-S. Chen, T.-H. Chao, *et al.*, "Low levels of high-density lipoprotein cholesterol in patients with atherosclerotic stroke: a prospective cohort study," *Atherosclerosis,* vol. 228, pp. 472-477, 2013.

[30] S. C. Kofoed, M.-L. M. Grønholdt, J. Bismuth, J. E. Wilhjelm, H. Sillesen, and B. G. Nordestgaard, "Echolucent, rupture-prone carotid plaques associated with elevated triglyceride-rich lipoproteins, particularly in women," *Journal of vascular surgery,* vol. 36, pp. 783-792, 2002.

[31] B. G. Nordestgaard, M.-L. M. Grønholdt, and H. Sillesen, "Echolucent rupture-prone plaques," *Current opinion in lipidology,* vol. 14, pp. 505-512, 2003.

[32] G. Sarlon-Bartoli, A. Boudes, C. Buffat, M. Bartoli, M. Piercecchi-Marti, E. Sarlon, *et al.*, "Circulating lipoprotein-associated phospholipase A2 in high-grade carotid stenosis: a new biomarker for predicting unstable plaque," *European Journal of Vascular and Endovascular Surgery,* vol. 43, pp. 154-159, 2012.

[33] D. Mannheim, J. Herrmann, D. Versari, M. Gössl, F. B. Meyer, J. P. McConnell, *et al.*, "Enhanced expression of Lp-PLA2 and lysophosphatidylcholine in symptomatic carotid atherosclerotic plaques," *Stroke,* vol. 39, pp. 1448-1455, 2008.

[34] H.-H. S. Oei, I. M. Van Der Meer, A. Hofman, P. J. Koudstaal, T. Stijnen, M. M. Breteler, *et al.*, "Lipoprotein-associated phospholipase A2 activity is associated with risk of coronary heart disease and ischemic stroke: the Rotterdam Study," *Circulation,* vol. 111, pp. 570-575, 2005.

[35] M. S. Elkind, W. Tai, K. Coates, M. C. Paik, and R. L. Sacco, "Lipoprotein-associated phospholipase A2 activity and risk of recurrent stroke," *Cerebrovascular Diseases,* vol. 27, pp. 42-50, 2009.

[36] K. B. Walsh, K. Hart, S. Roll, M. Sperling, D. Unruh, W. S. Davidson, *et al.*, "Apolipoprotein AI and paraoxonase-1 are potential blood biomarkers for ischemic stroke diagnosis," *Journal of Stroke and Cerebrovascular Diseases,* vol. 25, pp. 1360-1365, 2016.

[37] H. Dong, W. Chen, X. Wang, F. Pi, Y. Wu, S. Pang, *et al.*, "Apolipoprotein A1, B levels, and their ratio and the risk of a first stroke: a meta-analysis and case–control study," *Metabolic brain disease,* vol. 30, pp. 1319-1330, 2015.

[38] D. C. Chan, J. Pang, B. McQuillan, J. Hung, J. P. Beilby, P. H. R. Barrett, *et al.*, "Plasma proprotein convertase subtilisin kexin type 9 as a predictor of carotid atherosclerosis in asymptomatic adults," *Heart, Lung and Circulation,* vol. 25, pp. 520-525, 2016.

[39] K. Gasbarrino, C. Mantzoros, J. Gorgui, J. P. Veinot, C. Lai, and S. S. Daskalopoulou, "Circulating chemerin is associated with carotid plaque instability, whereas resistin is

related to cerebrovascular symptomatology," *Arteriosclerosis, thrombosis, and vascular biology,* vol. 36, pp. 1670-1678, 2016.

[40] L. A. Saarikoski, R. K. Huupponen, J. S. Viikari, J. Marniemi, M. Juonala, M. Kähönen, *et al.*, "Adiponectin is related with carotid artery intima-media thickness and brachial flow-mediated dilatation in young adults—the Cardiovascular Risk in Young Finns Study," *Annals of medicine,* vol. 42, pp. 603-611, 2010.

[41] J. Gorgui, K. Gasbarrino, M. K. Georgakis, M. A. Karalexi, B. Nauche, E. T. Petridou, *et al.*, "Circulating adiponectin levels in relation to carotid atherosclerotic plaque presence, ischemic stroke risk, and mortality: a systematic review and meta-analyses," *Metabolism,* vol. 69, pp. 51-66, 2017.

[42] J. Gairolla, R. Kler, M. Modi, and D. Khurana, "Leptin and adiponectin: pathophysiological role and possible therapeutic target of inflammation in ischemic stroke," *Reviews in the Neurosciences,* vol. 28, pp. 295-306, 2017.

[43] S. Holm, T. Ueland, T. B. Dahl, A. E. Michelsen, M. Skjelland, D. Russell, *et al.*, "Fatty Acid binding protein 4 is associated with carotid atherosclerosis and outcome in patients with acute ischemic stroke," *PLoS One,* vol. 6, p. e28785, 2011.

[44] J. Jia, A. Wang, J. Wang, J. Wu, X. Yan, Y. Zhou, *et al.*, "Homocysteine and its relationship to asymptomatic carotid stenosis in a Chinese community population," *Scientific reports,* vol. 6, pp. 1-7, 2016.

[45] S. Alsulaimani, H. Gardener, M. S. Elkind, K. Cheung, R. L. Sacco, and T. Rundek, "Elevated homocysteine and carotid plaque area and densitometry in the Northern Manhattan Study," *Stroke,* vol. 44, pp. 457-461, 2013.

[46] J.-M. Davaine, T. Quillard, R. Brion, O. Lapérine, B. Guyomarch, T. Merlini, *et al.*, "Osteoprotegerin, pericytes and bone-like vascular calcification are associated with carotid plaque stability," *PLoS One,* vol. 9, p. e107642, 2014.

[47] A. Schiro, F. L. Wilkinson, R. Weston, J. V. Smyth, F. Serracino-Inglott, and M. Y. Alexander, "Elevated levels of endothelial-derived microparticles and serum CXCL9 and SCGF-β are associated with unstable asymptomatic carotid plaques," *Scientific reports,* vol. 5, pp. 1-12, 2015.
